# Individual Differences in Impulsivity Predict Anticipatory Eye Movements

**DOI:** 10.1371/journal.pone.0026699

**Published:** 2011-10-26

**Authors:** Laetitia Cirilli, Philippe de Timary, Phillipe Lefèvre, Marcus Missal

**Affiliations:** 1 Institute of Neurosciences (IoNS), Université Catholique de Louvain, Brussels, Belgium; 2 Department of Adult Psychiatry, Academic Hospital Saint-Luc, Brussels, Belgium; 3 Institute of Information and Communication Technologies, Electronics and Applied Mathematics (ICTEAM), Université catholique de Louvain, Louvain-la-Neuve, Belgium; University of Manchester, United Kingdom

## Abstract

Impulsivity is the tendency to act without forethought. It is a personality trait commonly used in the diagnosis of many psychiatric diseases. In clinical practice, impulsivity is estimated using written questionnaires. However, answers to questions might be subject to personal biases and misinterpretations. In order to alleviate this problem, eye movements could be used to study differences in decision processes related to impulsivity. Therefore, we investigated correlations between impulsivity scores obtained with a questionnaire in healthy subjects and characteristics of their anticipatory eye movements in a simple smooth pursuit task. Healthy subjects were asked to answer the UPPS questionnaire (*U*rgency *P*remeditation *P*erseverance and *S*ensation seeking Impulsive Behavior scale), which distinguishes four independent dimensions of impulsivity: Urgency, lack of Premeditation, lack of Perseverance, and Sensation seeking. The same subjects took part in an oculomotor task that consisted of pursuing a target that moved in a predictable direction. This task reliably evoked anticipatory saccades and smooth eye movements. We found that eye movement characteristics such as latency and velocity were significantly correlated with UPPS scores. The specific correlations between distinct UPPS factors and oculomotor anticipation parameters support the validity of the UPPS construct and corroborate neurobiological explanations for impulsivity. We suggest that the oculomotor approach of impulsivity put forth in the present study could help bridge the gap between psychiatry and physiology.

## Introduction

Impulsivity describes one's tendency to act without forethought. It is a personality trait that profoundly influences one's behavior and can be an indicator of the development of several psychiatric diseases [1]. To organize behavior and allow some degree of anticipation of future events, the brain needs to make predictions based on information received from sensory organs. From these predictions emerges a representation of how one's action may influence the world [2]. We believe therefore that impulsive actions and abnormal behavior might be due to the inability of individuals to set up predictions normally. A specific example of the brain making predictions based on the repetition of a task is the generation of anticipatory eye movements in response to a moving target. If impulsivity is indeed connected with the basic neurological processes underlying prediction, we expect that it should be correlated with anticipatory eye movements that specifically depend on prediction. Thus, the aim of this paper is to study the relationship between impulsivity and anticipatory eye movements, i.e. saccades and smooth pursuit.

The UPPS [3] is a well validated and frequently used questionnaire that distinguishes four independent dimensions of impulsivity: Urgency, *lack of* Premeditation, *lack of* Perseverance, and Sensation seeking. The first three dimensions describe deficits related to “*negative*” aspects of impulsivity: the difficulty to restrain behavioral reactions in situations that elicit strong emotion (Urgency), the difficulty to anticipate expected situations (*lack of* Premeditation), and the difficulty to sustain prolonged, enduring activity (*lack of* Perseverance). The fourth dimension, sensation seeking, is a “*positive*” dimension that describes the tendency to search for new, highly emotionally-arousing situations. Besides being strongly related to pathology [1], impulsivity (particularly *lack of* premeditation) has also been related to deficits in decision making [4] as measured by choice tasks such as the Iowa Gambling task, and even to the depreciation of rewards as measured by delay discounting tasks [5]-[7]. However, these tasks test slow, complex cognitive processes. So far, the possibility that impulsivity could be related to basic, low level neural mechanisms has never been examined. In this study, we tested the possibility that impulsivity is related to standard oculomotor measures such as the latency and speed of anticipatory eye movements. One dimension of impulsivity in particular, the lack of premeditation, is by definition a difficulty in anticipating future events. We therefore expect that it could be well-correlated with the anticipatory oculomotor measures.

Anticipation has often been studied using eye movements as a tool. Indeed, saccadic and smooth anticipatory eye movements have been well described in humans and other primates [8]–[21], and the role of frontal structures involved in their control has been partly elucidated [22]–[26]. The interest of testing whether the characteristics of anticipatory movements correlate with UPPS scores is twofold. Firstly, it would help to understand the idiosyncratic differences in oculomotor anticipation between humans. Indeed, it has been often observed that there are large variations between humans in their capacity to initiate anticipatory movements [13]; M. Missal, personal observation). Some human healthy subjects are good anticipators, others are not. Secondly, finding a correlation would relate a personality trait commonly described in psychiatry with a well characterized, objectively measurable behavior, whereas questionnaires on personality may be subject to personal biases. Specific facets of impulsivity could be sustained by the same neurophysiological processes known to modulate anticipatory eye movements, involving the cortical-basal ganglia oculomotor loop. Indeed, the basal ganglia and the dopamine are dysregulated in impulsive subjects [7], [27]–[29]. Therefore, observed correlations would suggest that this important personality trait could be related to physiological mechanisms that are responsible for the development of anticipation, i.e. setting up predictions.

Two hypotheses might characterize the relationship between impulsivity and anticipation. Impulsivity could be due to a general state of increased arousal. We would expect in that case more frequent and faster anticipatory responses in impulsive subjects. On the other hand, impulsivity could be due to disruption of neurophysiological processes that underlie predictive mechanisms necessary for anticipation. We would expect on the contrary less frequent or slower anticipatory eye movements in subjects presenting with impulsivity or lack of premeditation.

## Methods

### Participants

Twenty-four healthy subjects were tested in the present study. All subjects gave written informed consent. The Biomedical Ethics Committee of Université catholique de Louvain approved the study (N/Ref: 2010/08/MAR/078; N° Belgian record: B40320108375). The mean age of the group was 47.0±1.9 years (with 54% men). The participants were recruited through advertisement. We inquired about past or present neurological or psychiatric disorders which were exclusion criteria. All the subjects also filled out a socio-demographic questionnaire. No IQ scores were available. Our population studied 15.2±0.5 years in school and the majority of our sample worked as qualified employees.

Subjects were seated in a darkened room facing a dimly-lit projection screen (Barco, Kortrijk, Belgium). The viewable screen area measured 195×146 cm, the resolution was 800×600 pixels and the refresh rate was 100 Hz. Their heads rested on a chin-rest 150 cm from the screen. Eye movements were recorded at 1000 Hz using an EyeLink 1000 fixed camera (SR Research Ltd., Mississauga, Ontario, Canada).

### Visual stimulus

**Figure 1:**
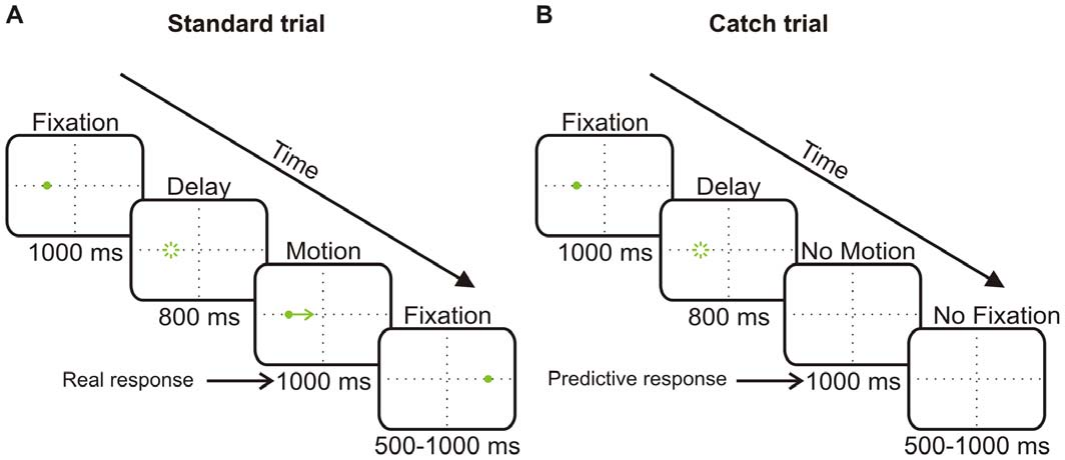
Experimental paradigm. A spot target appeared slightly offset to the left or right of the screen (first panel) and was fixated by the subject. The target disappeared during the delay period (second panel). After the delay in standard trials (A), the spot reappeared moving towards the center of the screen (third panel). It came to rest on the opposite side for a random amount of time (fourth panel). Only the spot was visible on the screen; the horizontal and vertical dashed lines are shown here for reference. The numbers below the panels indicate the duration of each interval. For catch trials (B), the spot appeared only during the first fixation and did not reappear after the delay until the next trial.

The visual pursuit stimulus consisted of a 0.75 deg green dot with a luminance of 0.5 cd/m^2^ appearing on a black background. During a trial, the target first appeared at a position offset by 7 deg either to the right or to the left of the center of the screen. After a fixation period of 1000 ms, the target disappeared, indicating the start of the delay interval. The delay lasted 800 ms, after which in 75% of trials (Standard trials) the target reappeared, offset by an additional 3 deg towards the periphery (total offset  = 10 deg; step-ramp ‘Rashbass’ stimulus; [30] and immediately started to move towards the center of the screen at a velocity of 25 deg/s (Figure 1). Total target excursion was 25 deg. Upon reaching the end of its trajectory, the target stopped and remained immobile for a random amount of time (500, 750 or 1000 ms) before disappearing again, marking the end of the trial. In 25% of trials (Catch trials) the target did not reappear after the delay. Instead the blank screen persisted for the remainder of the trial. The inter-trial interval was 1000 ms. In all blocks, subjects were asked to follow the moving target with their eyes as accurately as possible. The experimental session consisted of at least 16 blocks of 50 trials each. Each block lasted 5 minutes. Between 800 and 900 trials were collected from each subject.

### Eye movements

The EyeLink eyetracker recorded pupil position directly. Subsequent analysis was performed in MATLAB (The MathWorks Inc., Natick, Mass.) using in-house software. Horizontal and vertical eye position were digitally differentiated and filtered (25 Hz cutoff) to compute eye velocity. Anticipation onset was detected using the algorithm of de Hemptinne et al. [31]. Filtered velocity was required to exceed a threshold of 1.5 deg/s for at least 100 ms to meet the requirement of anticipation. The threshold was determined empirically depending on the level of eye signal noise during the fixation period (around ±1°/s; see [31]).

The dependent variables measured were: eye velocity at the time of target motion onset (with a de-saccaded interpolation; [32], referred to as ‘anticipatory eye velocity’,), the latency of smooth pursuit after target motion onset (‘visual pursuit latency’, predictive visual pursuit in the case of catch trials). We measured also the latency and the amplitude of the first anticipatory saccade during the delay period, the gain of standard trials (gain  =  eye velocity/target velocity) and the number of anticipatory saccade trials as a percentage.

### UPPS Impulsive Behavior Scale

The UPPS impulsivity scale includes 45 items and measures four distinct personality dimensions of impulsive behavior, including Urgency (12 items), *lack of* Premeditation (11 items), *lack of* Perseverance (10 items), and Sensation Seeking (12 items). Each item is rated on a 1 =  “not at all to” 4 =  “very much” – point scale. Each subscore is obtained by summing values selected by the subject for all questions related to the particular dimension. The total UPPS score is the sum of all subscores. The subscales demonstrated good internal consistencies in the original (Cronbach α, range from .82 to .91); [3] and in the present study (Cronbach α, range from .72 to .93).

Half of the subjects completed the French version of the UPPS questionnaire [33] (see Table S1) before the oculomotor experiment and the other half after. No difference between these two groups was observed. In order to compare pursuit characteristics between subgroups that differed as widely as possible in impulsivity, we selected the 8 subjects with the lowest scores (total UPPS score <77) and the 8 with the highest (total UPPS score >87) for several analyses (see Results).

### Statistical analysis

Statistical analyses were performed with Statistica 7.1 (StatSoft Inc.) Nonparametric Spearman-rank correlation tests were used to assess the relationship between characteristics of eye movements and the UPPS scores at a 5% confidence level. Mann-Whitney nonparametric tests were used to compare the characteristics of eye movements between high and low impulsive subgroups.

## Results

### UPPS scores of impulsivity

The mean of total UPPS score and the mean of the four subscores obtained (urgency, *lack of* premeditation, *lack of* perseverance and sensation seeking) for the 24 experimental subjects were 84±4, 24±2, 19±1, 18±1, 23±1, respectively. Observed scores were in the range observed for healthy subjects of that age group [3].

### Impulsivity and anticipatory eye movements

**Figure 2:**
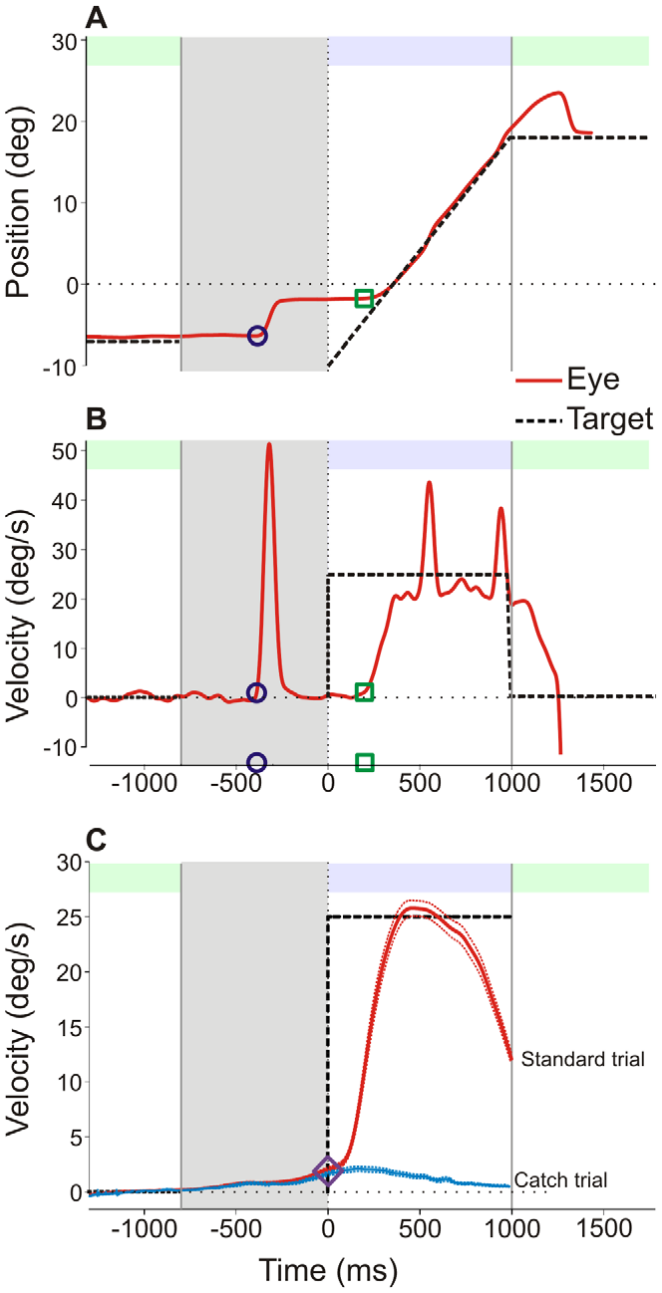
Example of one standard trial and mean velocities. A & B. Example of one standard trial in one subject with an anticipatory saccade (AS response) showing A: eye and target position traces and B: corresponding eye and target velocities. Anticipation onset is marked with a blue circle and visual smooth pursuit latency with a green square. C: Desaccaded anticipatory eye velocity profile (mean ± sem) for standard and catch trials averaged for n = 24 subjects. Anticipatory eye velocity at target onset is shown with a purple diamond. On each panel, the grey area represents the delay period (800 ms). On the top of each panel, the green areas are the fixation periods (fixation 1: 1000 ms and fixation 2: randomized 500, 750 or 1000 ms). The light purple area is the interval of target motion (1000 ms).

A total of 12 081 pursuit trials were recorded (n = 24 subjects). Figure 2 shows a typical example of an observed response during the oculomotor task. During the delay period (*gray area*), the anticipatory response was initiated with a saccade (referred to as ‘anticipatory saccade response’ or ‘AS’). Pursuit latency after target motion onset was∼120 ms on average (indicated with a square).

**Figure 3:**
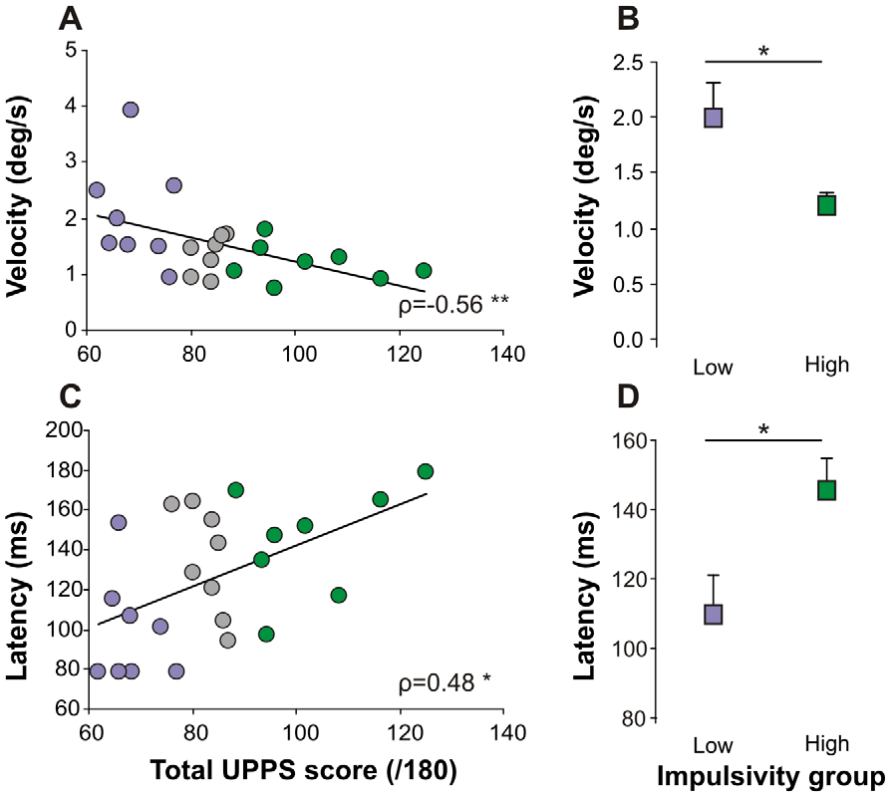
Correlations between impulsivity total score and anticipatory eye movements. A. Significant correlation between the anticipatory eye velocity at the target onset and the total UPPS score in our healthy experimental group (n = 24, median). B. Comparison of the anticipatory eye velocity (median + sem) at the target onset between High (n = 8) and Low (n = 8) impulsive subgroups. C. Significant correlation between the visual smooth pursuit latency and the total UPPS score in our healthy experimental group (n = 24, median). D. Comparison of the visual smooth pursuit latency (median + sem) between High (n = 8) and Low (n = 8) impulsive subgroups. The Spearman coefficient ρ is indicated. Statistics are * p≤0.05 and ** p≤0.01. The color code for the high and low impulsive subgroups is green and purple respectively.

In this study, we hypothesized that impulsivity could be correlated with anticipatory oculomotor behavior. Therefore, we tested whether characteristics of anticipatory responses could be correlated with the total UPPS score and the four different subscores. We found that some characteristics of anticipatory responses were indeed correlated with UPPS scores except the anticipatory saccade latency, the percentage of anticipatory saccade trials and the gain, which are not significantly correlated to any of the UPPS total score or subscores. A higher total UPPS score was negatively correlated with anticipatory eye velocity (Figure 3A) and was positively correlated with visual pursuit latency (Figure 3C). Moreover, when low and high impulsivity subgroups were compared, significant differences in movement characteristics were also observed (Figure 3B and 3D). High total UPPS subjects had lower anticipatory eye velocity and longer visual pursuit latencies.

This first analysis shows that impulsivity scores are well correlated with oculomotor behavior. Furthermore, it suggests that idiosyncratic variations of anticipation can be partly attributed to impulsivity.

**Figure 4:**
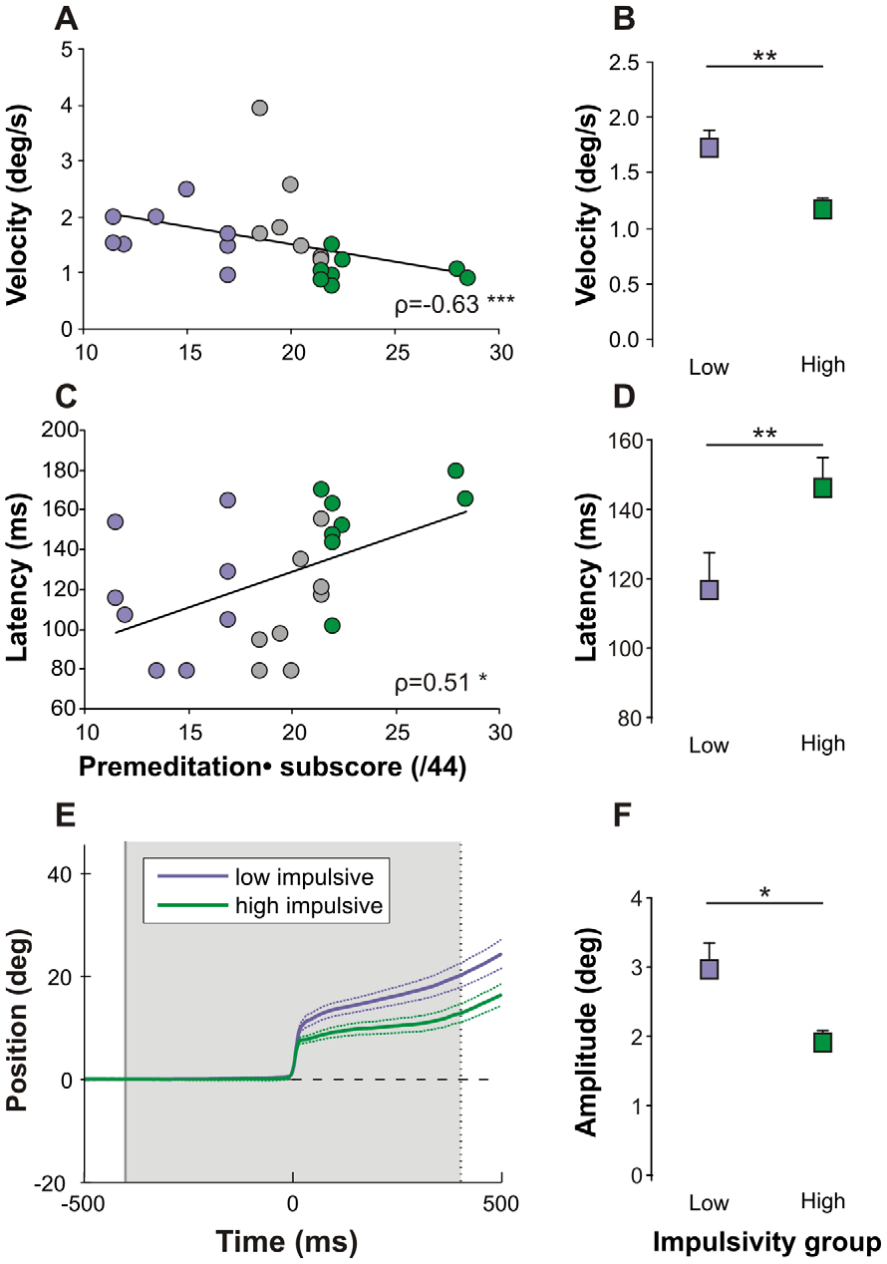
Correlations between *lack of* premeditation subscore of impulsivity and anticipatory eye movements. A. Significant correlation between the anticipatory eye velocity at the target onset and the *lack of* premeditation subscore in our healthy experimental group (n = 24, median). B. Comparison of the anticipatory eye velocity at the target onset (median + sem) between High (n = 8) and Low (n = 8) impulsive subgroups. C. Significant correlation between the visual smooth pursuit latency and *lack of* premeditation subscore in our healthy experimental group (n = 24, median). D. Comparison of the visual smooth pursuit latency (median + sem) between High (n = 8) and Low (n = 8) impulsive subgroups. E. Eye positions (median ± sem) of high and low impulsive subgroups during the delay period (800 ms). F. Comparison of the amplitude of anticipatory saccades (median ± sem) between High (n = 8) and Low (n = 8) impulsive subgroups. The Spearman coefficient ρ is indicated. Statistics are * p≤0.05; ** p≤0.01 and *** p≤0.001. The color code for the high and low impulsive subgroups is green and purple respectively.

Table 1 shows correlations between UPPS scores and anticipatory eye velocity and visual pursuit latency. Strong correlations (p<0.01, **) were found with the total UPPS score and *lack of* premeditation. Other subscores were weakly correlated with the same characteristics (p<0.05, *). Although a trend is observed for the correlation with AS amplitude, these results suggest that the *lack of* premeditation (lack of forethought) could be related to idiosyncratic differences in anticipatory oculomotor behavior. Figure 4 shows the correlations observed between *lack of* premeditation and anticipatory eye velocity (Figure 4A) and visual pursuit latency (Figure 4C). Lack of premeditation was linked with a *lower* anticipatory velocity (negative correlation) and *longer* pursuit latency (positive correlation). This result was further strengthened by comparing the two extreme subgroups of subjects (low and high impulsive, see Methods) as shown on Figure 4B (velocity) and 4D (latency). Highly impulsive subjects had lower anticipatory eye velocity and longer visual pursuit latencies. Similarly, saccadic amplitude was smaller in high impulsive subjects than in low impulsive subjects (Figure 4E and 4F). These results show that contrary to our first hypothesis, increased impulsivity does not lead to faster or larger responses in healthy subjects.

**Table 1:** Significant correlations of eye movement parameters of saccadic anticipation trials with UPPS score and subscores.

	Eye movements parameters		
UPPS scores	Velocity (deg/s)	Latency (ms)	AS amplitude (deg)
Total	ρ = −0.56 **	ρ = 0.48 *	ns
Urgency	ns	ρ = 0.41 *	ns
• Premeditation	ρ = −0.63 ***	ρ = 0.51 *	ρ = −0.37 ( 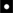 p = 0.074)
• Perseverance	ρ = −0.42 *	ρ = 0.44 *	ns
Sensation seeking	ρ = −0.43 *	ns	ns

The median of anticipatory eye velocity and visual pursuit latency are used. The Spearman coefficient ρ is indicated. Statistics are * p≤0.05; ** p≤0.01 and *** p≤0.001, 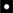 indicated a tendency p≤0.1 . Abbr. • indicates that the score referred to a ‘*lack of’.* AS: anticipatory saccade.

### Standard versus catch trials

**Figure 5:**
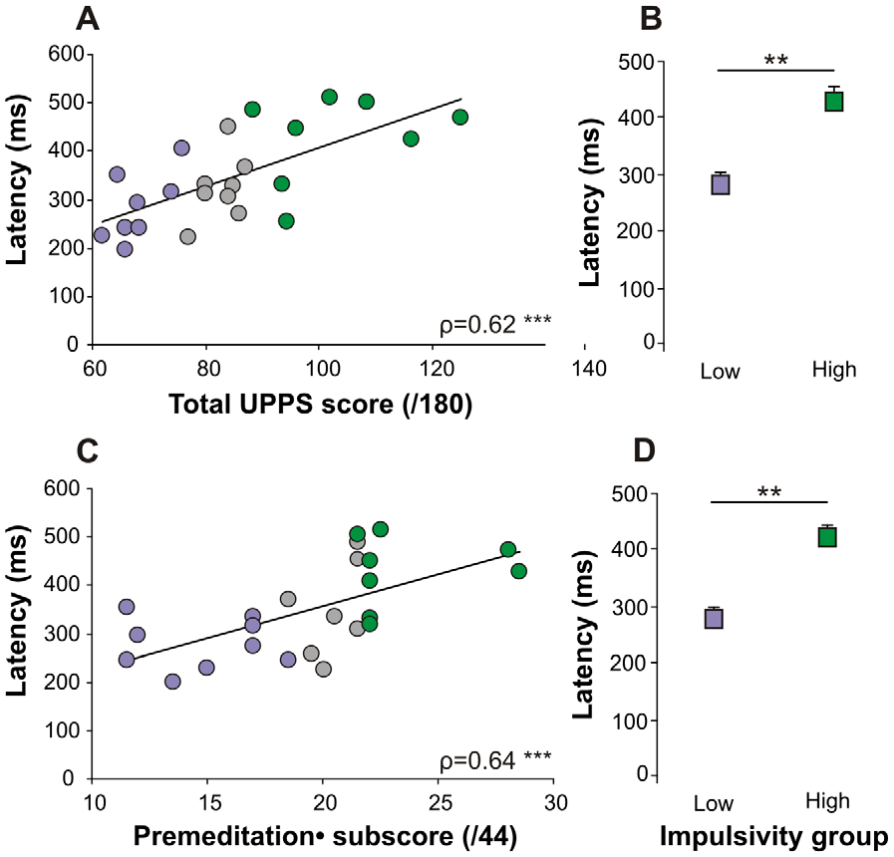
Correlations between total score and *lack of* premeditation subscore of impulsivity and anticipatory eye movements in CATCH trials. A. Significant correlation between the “*predictive*” smooth pursuit latency and the total UPPS score in our healthy experimental group (n = 24, mean). B. Comparison of the “*predictive*” smooth pursuit latency (mean + sem) between High (n = 8) and Low (n = 8) impulsive subgroups. C. Significant correlation between the “*predictive*” smooth pursuit latency and *lack of* premeditation subscore in our healthy experimental group (n = 24, mean). D. Comparison of the “*predictive*” smooth pursuit latency (mean + sem) between High (n = 8) and Low (n = 8) impulsive subgroups. The Spearman coefficient ρ is indicated. Statistics are ** p≤0.01 and *** p≤0.001. The color code for the high and low impulsive subgroups is green and purple respectively.

It has been shown previously that the initial eye acceleration after target motion onset that is commonly referred to as ‘visual’ pursuit latency is in fact still largely influenced by anticipation [34]. Indeed, the anticipatory pursuit response continues and blends with the visually guided pursuit response after target motion onset. During catch trials, no visual pursuit target was displayed at the end of the delay period. Therefore, eye movements after the time of expected target motion onset in catch trials can only be of an anticipatory or predictive nature. In order to isolate the predictive component of pursuit initiation after target motion onset, 25% of catch trials were randomly interleaved with standard trials (see Methods). Figure 2C shows a comparison between eye velocity in standard (*red traces*) and catch trials (*blue traces*). The latency of the smooth pursuit response in the absence of the visual target in catch trials will be referred to as ‘predictive pursuit latency’ (see Table 2). During catch trials, the strongest correlations were found between the subscore of *lack of* premeditation and predictive pursuit latency (positive Spearman ‘Rho’; see Figure 5). Only a trend was noticed for the correlation between AS amplitude and *lack of* premeditation. We conclude that the UPPS score of *lack of* premeditation could directly be correlated with prediction in the oculomotor domain.

**Table 2:** Significant correlations of eye movement parameters of saccadic anticipation CATCH trials with UPPS score and subscores.

	Eye movements parameters		
UPPS scores	Velocity (deg/s)	Latency (ms)	AS amplitude (deg)
Total	ρ = −0.53 **	ρ = 0.62 ***	ns
Urgency	ns	ρ = 0.53 **	ns
• Premeditation	ρ = −0.58 **	ρ = 0.64 ***	ρ = −0.40 ( 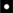 p = 0.054)
• Perseverance	ρ = −0.55 **	ρ = 0.48 *	ns
Sensation seeking	ns	ρ = 0.46 *	ns

The mean of anticipatory eye velocity and “*predictive*” pursuit latency are used. The Spearman coefficient ρ is indicated. Statistics are * p≤0.05; ** p≤0.01 and *** p≤0.001, 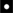 indicated a tendency p≤0.1 . Abbr. • indicates that the score referred to a ‘*lack of’.* AS: anticipatory saccade.

## Discussion

Impulsivity is an important personality trait that describes tendencies to act without forethought. It has been related to specific periods of life such as adolescence [35]–[36], to severe personality disorders such as borderline or psychopathic personality, as well as to several externalizing psychiatric disorders, for instance mania, substance abuse or dependence, bulimia nervosa, ADHD and other conduct disorders [1]. Furthermore, “impulsivity” is, after “subjective distress” the most common diagnostic criteria for psychiatric illnesses in the fourth version of the Diagnostic and Statistical Manual of Mental Disorders (DSM IV, [37]. In the aim of identifying different dimensions of impulsivity, Whiteside and Lynam [3] have administered the five factor model of personality and several impulsivity questionnaires to 400 young adults. Factorial analysis identified different facets, from which they constructed the UPPS questionnaire, now an excellent tool for studies on impulsivity.

Our study is one of the first to show a significant relationship between impulsivity as measured by UPPS scores and simple oculomotor predictive behavior. The strongest and most often observed correlations in our data were found between the score of *lack of* premeditation and anticipation characteristics. Premeditation can best be described as the tendency to ‘think and reflect on the consequences of an act before engaging in that act’ [38]. More precisely, we found that scores of *lack of* premeditation were negatively correlated with velocity and positively correlated with latency. *Lack of* premeditation has already been found to be a predictor of poor decision making using the complex procedure of the Iowa Gambling Task [4]. Our data supports that it is also associated with basic markers of anticipation, i.e. a lower anticipatory pursuit velocity and a longer latency.

This study shows how anticipatory pursuit could be useful for further understanding the neuronal basis of premeditation in humans. It is indeed a unique tool to investigate the functionality of these cortical-basal ganglia loops that, although of great functional importance, cannot be explored by classical electrophysiological approaches. We suggest that the approach initiated in the present study might be particularly fruitful for bridging the gap between psychiatry and fundamental neurosciences. Indeed, although our understanding of the processes underlying impulsivity has been much improved using the carefully designed approach of Whiteside and Lynam [3], it still relies on verbal answers to questions that are subject to interpretation and strategies that might influence the final results. The UPPS is a written questionnaire, and not all subjects are able to accurately estimate their own personality. Therefore, answers to questions are likely subject to personal biases and misinterpretations, whereas eye movement measures of impulsivity should be free from subjective biases.

As suggested by Evenden [58], the understanding of the neurobiological mechanisms underlying psychological or psychiatric phenomena still lacks good experimental data. Furthermore, neuronal processes involved in impulsivity are extremely complex and poorly understood (see [38], for review). Our observation of discrete, specific correlations between distinct UPPS factors and visual pursuit anticipation parameters, in particular between *lack of* premeditation and different parameters in saccadic anticipation responses, supports the validity of the UPPS construct but also suggests neurobiological explanations for impulsivity, that involve functional loops between subcortical and cortical areas.

Our observations also provide a new perspective for understanding premeditation, a factor that has also been correlated with deficits in decision making but whose automatic or voluntary nature has been debated. Whereas animal models may allow a simultaneous electrophysiological measurement of the activity of the striatum and the frontal cortex during a learning task, such a procedure is not applicable to human beings, for obvious ethical reasons. However, one fairly direct way of studying the activity of the corticostriatal loop is the measurement of anticipatory eye movement during an oculomotor task. Indeed, anticipatory saccadic and smooth pursuit eye movements have extensively been studied in primates [21]; [16]–[17], [20], [78]–[79]; [10]; [11]; [14]; [13]; [9]; [12]; [15]; [8] and involve both basal ganglia [80]–[81]; [82]; [46]; [83], [84]; [85]; [86], [87]–[88], [89]; [90]; [91] and frontal cortex [24]; [23]; [26]; [22]; [25]. Neuronal processes driving eye movements have been studied in detail and, although some unanswered questions still persist, they are among the best described neural systems. Therefore, correlations between UPPS scores and pursuit characteristics are particularly important, and support this type of approach in personality evaluation. The existence of such a link between neurophysiology and psychiatry may find interesting applications in clinical environments.

Furthermore, oculomotor predictive behavior reflects the function of anatomical loops between the frontal cortex and basal ganglia, via the thalamus. It is possible therefore that these loops do not only reflect motor or oculomotor activity but also serve as substrates for cognitive or emotional functions, which play a role in impulsive behavior and other psychiatric conditions [39]–[44]. Indeed, in humans, anticipatory eye movements were shown to rely on frontal cortex [22], [24], [45]–[49] and basal ganglia [40], [50]. These cerebral structures play also a key role in decision making [38], [51] and impulsivity as shown by the famous Phineas Gage case and others clinical/lesion or psychiatric evidences [46], [52]–[54]. Dopamine (DA), the key neurotransmitter of these functional loops, has also been theorized to play an important role in impulsivity [27], [55]–[57], but the precise mechanisms involved remain unclear so far. Schultz, Tremblay and Hollerman [2] suggested that to organize behavior, the brain needs to make predictions from information received from sensory organs, to allow to some extent the anticipation of future events. They ascribed a central role to the corticostriatal loops in setting up these predictions. Indeed, in animal models, DA neurons of the striatum and frontal cortex show phasic responses to stimuli during procedural learning of specific behavioral tasks including rewarding tasks or conditional associative learning in electrophysiological studies in monkeys [59]–[60]; [61]–[63]; [64]; [65]–[66]; [2] and in fMRI studies in humans [67]; [68]. Our observation of correlations between the *lack of* premeditation and blunted anticipatory pursuit characteristics suggests that deficits in predictions and suboptimal functioning of the corticostriatal loops in setting up those predictions [2] may be a central mechanism involved in the development of impulsive behavior. This would also be consistent with the recent observation of decreased midbrain D2/D3 autoreceptor availability and an abnormal dopamine network in impulsive subjects [27]. Dopaminergic neurons initially respond during consumption of unexpected rewards, but eventually fire in response to reward-predicting cues and show decreased activity when expected rewards are omitted (for reviews, see [69]; [61]–[63]). During conditioning, phasic DA responses appear to encode predictions about future events, either via an explicit reward prediction-error signal [61]–[63], [70]; [71]; [72], or by a more generic signal for learning action-perception contingencies [73]; [74]. Dopamine, the neurotransmitter involved in these corticostriatal loops, plays an important role in enhancing learning by modifying neuronal activity in the corticostriatal loops associated with a major stimulus or reward (for reviews see [75]; [76]–[77]). Based on these findings, it has been suggested that activity in this circuit supports various forms of reward prediction, reinforcement-based learning and approach-related behavior. This suggests a role of both striatum and frontal cortex and of their interaction in the setting up of predictions. The importance of anatomical loops between the frontal cortex and basal ganglia, via the thalamus, so-called corticalstriatal frontal loops has been well supported by several lines of evidence and are involved in impulsive behavior [27].

In summary, higher scores of impulsivity in healthy subjects were correlated with *smaller* anticipatory saccades, *longer* smooth pursuit latencies and *lower* anticipatory pursuit velocities, contrary to what might be expected from the general understanding of ‘impulsivity’ that is often associated with faster responses. Our results suggest that the cognitive processing of information needed to anticipate future events is affected by impulsivity, resulting in delayed and slowed movements. In other words, if impulsivity may be defined as acting without forethought, our study suggests that anticipatory eye movements, which reflect the functioning of subcortical-cortical functional loops may provide us with clues on what happens during forethought.

## Supporting Information

Table S1 **Items in UPPS impulsive behavior scale.** From: Whiteside SP & Lynam DR. Personality and Individual Differences 30 (2001) 669–689. And French validation from: Van der Linden M et al., European Journal of Psychological Assessment 2006; Vol. 22(1):38–42.
(DOC)
