# Supplementary material for: Individual Differences in Impulsivity Predict Anticipatory Eye Movements
Source: PLoS One. 2011 Oct 26;6(10):e26699. doi: 10.1371/journal.pone.0026699 (PMC3202566; doi:10.1371/journal.pone.0026699)
Supplement: Table S1 — Items in UPPS impulsive behavior scale. From: Whiteside SP & Lynam DR. Personality and Individual Differences 30 (2001) 669–689. And French validation from: Van der Linden M et al., European Journal of Psychological Assessment 2006; Vol. 22(1):38–42. (DOC) [file pone.0026699.s001.doc]

***Supporting Information table S1***

*Items in UPPS impulsive behavior scale*

**Urgency**

1. I have trouble controlling my impulses.

2. I have trouble resisting my cravings (for food, cigarettes, etc.).

3. I often get involved in things I later wish I could get out of.

4. When I feel bad, I will often do things I later regret in order to make myself feel better now.

5. Sometimes when I feel bad, I can't seem to stop what I am doing even though it is making me feel worse.

6. When I am upset I often act without thinking.

7. When I feel rejected, I will often say things that I later regret.

8. It is hard for me to resist acting on my feelings.

9. I often make matters worse because I act without thinking when I am upset.

10. In the heat of an argument, I will often say things that I later regret.

11. I am always able to keep my feelings under control. * (R)

12. Sometimes I do things on impulse that I later regret

**Premeditation (*lack of*)**

1. I have a reserved and cautious attitude toward life.

2. My thinking is usually careful and purposeful.

3. I am not one of those people who blurt out things without thinking.

4. I like to stop and think things over before I do them.

5. I don't like to start a project until I know exactly how to proceed.

6. I tend to value and follow a rational, ``sensible'' approach to things.

7. I usually make up my mind through careful reasoning.

8. I am a cautious person.

9. Before I get into a new situation I like to find out what to expect from it.

10. I usually think carefully before doing anything.

11. Before making up my mind, I consider all the advantages and disadvantages.

**Perseverance (*lack of*)**

1. I generally like to see things through to the end.

2. I tend to give up easily. *(R)

3. Unfinished tasks really bother me.

4. Once I get going on something I hate to stop.

5. I concentrate easily.

6. I finish what I start.

7. I'm pretty good about pacing myself so as to get things done on time.

8. I am a productive person who always gets the job done.

9. Once I start a project, I almost always finish it.

10. There are so many little jobs that need to be done that I sometimes just ignore them all. *(R)

**Sensation Seeking**

1. I generally seek new and exciting experiences and sensations.

2. I'll try anything once.

3. I like sports and games in which you have to choose your next move very quickly.

4. I would enjoy water skiing.

5. I quite enjoy taking risks.

6. I would enjoy parachute jumping.

7. I welcome new and exciting experiences and sensations, even if they are a little frightening and unconventional.

8. I would like to learn to fly an airplane.

9. I sometimes like doing things that are a bit frightening.

10. I would enjoy the sensation of skiing very fast down a high mountain slope.

11. I would like to go scuba diving.

12. I would enjoy fast driving.

* (R) ± indicates that the item is reverse-scored.

From: Whiteside SP & Lynam DR. Personality and Individual Differences 30 (2001) 669-689.

And French validation from: Van der Linden M et al., European Journal of Psychological Assessment 2006; Vol. 22(1):38–42.
